# Reasons for presenteeism in different occupational branches in Sweden: a population based cross-sectional study

**DOI:** 10.1007/s00420-021-01701-2

**Published:** 2021-04-29

**Authors:** Staffan Marklund, Klas Gustafsson, Gunnar Bergström, Constanze Leineweber

**Affiliations:** 1 Department of Clinical Neuroscience, Division of Insurance Medicine, Karolinska Institutet, 171 77 Stockholm, Sweden; 2 Department of Occupational Health Sciences and Psychology, Centre for Musculoskeletal Research, University of Gävle, Gävle, Sweden; 3 Institute of Environmental Medicine, Unit of Intervention and Implementation Research for Worker Health, Karolinska Institutet, Stockholm, Sweden; 4 Stress Research Institute, Stockholm University, Stockholm, Sweden

**Keywords:** Presenteeism, Employment sector, Occupational branches, Cross sectional study

## Abstract

**Objective:**

To compare the prevalence and reasons for presenteeism in occupations in three branches defined as employees handling people, handling things or handling symbols.

**Method:**

A cross-sectional population-based cohort study was conducted. The study group was drawn from a representative sample (*n* = 6230) aged 16–64, who had been interviewed in 2015 or in 2017 for the Swedish Work Environment Surveys (SWES). The odds ratios (ORs) stratified by occupational category for reasons of presenteeism, with 95% confidence intervals (CI), were estimated using binomial multiple logistic regression analysis.

**Results:**

The study showed that presenteeism was more common among employees handling people (74%), when compared to employees handling things (65%) or handling symbols (70%). The most common reason for presenteeism among employees handling people was “I do not want to burden my colleagues”, while “Because nobody else can carry out my responsibilities” was most common in the other two categories. After control for socio-demography, work environments and health, the differences in reasons mostly remained significant between the three occupational categories.

**Conclusion:**

The differences between occupational categories are important for prevalence and reasons for presenteeism. As presenteeism affects the future health of employees and the productivity of the work unit, attempts to reduce presenteeism may be important. Because the reasons vary between occupations, customized preventive measures should be applied in different occupational settings. Among employees handling people, covering up for absence in work team is relevant, while among employees handling symbols and handling things the corresponding focus could be on shared responsibilities for specific tasks.

**Supplementary Information:**

The online version contains supplementary material available at 10.1007/s00420-021-01701-2.

## Introduction

As pointed out by several scholars, sickness presenteeism, that is, going to work despite illness, is related to the employees working conditions, to their health and to a number of other individual and organizational factors (Grinyer and Singleton 2000; Johns 2010; Karanika-Murray et al. 2015). Presenteeism has been shown to cause large costs for companies and for the society (Kinman 2019; Strömberg et al. 2017; Vänni et al. 2017), but has also been shown to increase the risk of future ill health (Aboagye et al. 2019; Gustafsson et al. 2019; Gustafsson and Marklund 2011, 2014). One large longitudinal study found for example that obesity and long-term health conditions were strongly associated with presenteeism (Keramat et al. 2020). Thus, it is essential to better understand the different reasons employees give for going to work despite illness.

A number of studies have found significant differences in the prevalence of presenteeism in occupational groups and or occupational sectors. Higher prevalence of presenteeism has been found among employees in the welfare, health and educational sectors compared to other sectors (Aronsson et al. 2000), among managers when compared employees without a management role (Hansen and Andersen 2008), among public sector employees when compared to private employed (Bockerman and Laukkanen 2010; Johansen et al. 2014), among blue collar employees when compared to white-collar employees (Gustafsson and Marklund 2011), and among self-employed when compared to employees (Nordenmark et al. 2019). One study showed that manual workers reported higher rates of presenteeism than non-manual workers, and that temporary workers reported higher presenteeism than permanent workers (Navarro et al. 2018). A review of presenteeism in different occupations in the health care sector showed that physicians reported higher rates of presenteeism than nurses and nursing assistants (Webster et al. 2019). Similarly, a study of employed in the educational sector found that teachers had a lower prevalence of presenteeism than other school employed (Dudenhöffer et al. 2017).

Fewer studies have been concerned with occupational differences in the reasons lying behind sickness presenteeism, and large share of them concerns occupations in the health sector. However, three studies have compared different occupational groups (Johansen et al. 2014; McKevitt et al. 1997; Navarro et al. 2018). In a study of mixed occupational groups Johansen et al. (2014) found large deviations in reasons between managers and non-managers, where the managers rarely gave economic reasons but often said that nobody else could carry out their obligation. In contrast, non-managers often reported that they could not afford taking sick leave or that they assumed negative effects of presenteeism on colleagues (Johansen et al. 2014). This finding finds support in a study of salaried workers that found that the most common reason for presenteeism was that they ‘did not want to be a burden to colleagues’ (Navarro et al. 2018). In another study comparing reasons for presenteeism among physicians and accountants, a majority of the physicians reported that they did not want to be unfair to colleagues, while accountants to a high degree reported that ‘no-one else could do the work’ (McKevitt et al. 1997). The concern among physicians that presenteeism would burden the colleagues was also mentioned in several other studies (Al Nuhait et al. 2017; Bracewell et al. 2010; Gustafsson Senden et al. 2016; Jena et al. 2012; Webster et al. 2019).

However, most of the conducted studies have only compared a few occupations and most of the studies on reasons for presenteeism concern hierarchical differences between occupations. There is a shortage of studies on horizontal differences in working conditions between occupational branches and how these may affect reasons for presenteeism, focusing on variations in the work object such as personal services versus industrial production.

To study work and social stratification, Kohn and Schooler introduced in 1983 a horizontal classification of occupations in three categories “working with things”, “working with data” and “working with humans” (Kohn and Schooler 1983). Similar horizontal classification systems have later been developed, although based on different theoretical grounds and different empirical sources. One study primarily concerned labor market inequalities (Blackburn et al. 2001; Lippa et al. 2014), while other have focused on work psychology and occupational perceptions to understand general occupational differences and gender differences in working conditions (Cerdas et al. 2019; Leijon et al. 2002; Lips-Wiersma et al. 2016).

The present study has adapted a classification similar to the one by Kohn and Schooler, based on differences and similarities regarding the object employees are working with (Kohn and Schooler 1983). The three categories have in the present study been named: “handling people”, “handling things” and “handling symbols” and were based on the Swedish version of the Statistical classification of economic activities in the European Community 2008 (EU 2008). The selection of occupations in each of the three categories will be presented in detail in the methods section.

Although there is no comprehensive theoretical model for presenteeism, there are a few rather similar suggested frameworks based on empirical experiences. Johns’ dynamic model (2010) includes contextual factors, health and person factors, where the contextual factors include work environment and work organization and person factors include attitudes to work as well as personality (Johns 2010). Lohaus and Habermann (2019) agree with Johns but divide the contextual factors into environmental, work-related and organizational factors. Keramat et al. (2020) on the other hand, exclude the contextual factors and focus on job factors, individual factors and health. As the present study is studying differences between occupational categories, the main theoretical assumption is that employees’ obligations at work are essential in their reported reasons for presenteeism. However, a number of other factors that previous studies have found important for the choice of going to work despite illness have also been used in the present study (Gustafsson et al. 2020; Johns 2010; Keramat et al. 2020; Lohaus and Habermann 2019). These are, age, sex, level of education, sector of employment, disposable income, physical and psychosocial work environment exposures and health complaints.

The present study had three aims. The first aim was to examine the prevalence of presenteeism, that is, going to work despite illness, among three occupational categories with varying work objects: handling people, handling things, and handling symbols. The second aim was to describe self-reported reasons for presenteeism in the three occupational categories. The third aim was to examine to what degree the differences in reasons for presenteeism between the three occupational categories remained after control for potential confounders.

## Methods

### Data sources and sample selection

Data were drawn from the Swedish Work Environment Surveys (SWES), (SWEA 2016) and the Longitudinal Integrated Database for Health Insurance and Labor Market studies (LISA) from Statistics Sweden (SCB 2019). The SWES surveys have been conducted every second year since 1989. The surveys are administered by Statistics Sweden to random samples of the Swedish employed population aged 16–64 through telephone interviews and a supplementary postal questionnaire. In the present study, data from 6230 individuals from the two separate SWES surveys of 2015 and 2017 were used, which were the first waves, where employees’ self-reported reasons for presenteeism were included. In the present study, the official translation of the survey questionnaire into English by Statistics Sweden has been used (www.scb.se). The survey covers data on a broad range of physical and psychosocial working conditions and health symptoms. The response rates for 2015 and 2017 were 66% and 65%, respectively. Information on occupation, background factors (age, sector, level of education sector of employment, branches, and disposable income), was derived from LISA (SCB 2019).

### Outcome variables

Self-reported *presenteeism* was measured in the SWES surveys of 2015 and 2017 by a single question (SWEA 2016) that has also been used in a number of previous Scandinavian studies (Aronsson et al. 2000; Gustafsson and Marklund 2011, 2014; Johansen et al. 2014). The question has the following wording:

“How many times during the past 12 months have you worked, even though you really should have not worked given your medical condition?”

The fixed four-point response scale was:

- Never (1)
- Once (2)
- Two to three times (3)
- Four times or more (4)

A test–retest of this question carried out by Demerouti and collaborators reported a reliability value of 0.58 (*p* < 0.01) (Demerouti et al. 2009).

Respondents who reported having been sickness present once or more were in the SWES surveys asked an additional question about their reasons. This question was used in a previous study on positive and negative reasons for presenteeism in Norway and Sweden (Johansen et al. 2014), but has not been tested for psychometric qualities.

“Why did you go to work although you were ill?”

The respondents were asked to select one or more of the 10 fixed response alternatives, including one, where they could give their own reason:

1. Because I do not want to burden my colleagues
2. Because nobody else is able to carry out my responsibilities
3. Because I enjoy my work
4. Because I can’t afford taking sick leave
5. Because I do not want to be considered lazy or unproductive
6. Because my pride depends on not taking sick leave
7. Because I am worried about being laid off
8. Because going to work was beneficial for my health
9. Because I want to maintain my social network
10. There were other reasons that I went to work

The last response alternative “There were other reasons that I went to work” was not used in this study. The reason for the response alternative “Because I can’t afford taking sick leave” was used in the survey, since the Swedish sickness insurance system does not compensate the first day off and is restricted to cover at best about 80% of the employees’ loss of income. Respondents could give several reasons and the responses do for this reason add up to more than 100 percent.

### Exposure variable

The main exposure variable was occupational category. With guidance from two Swedish studies, three occupational categories were formed on the basis of the work object of an occupation: handling persons, handling things, handling symbols (Cerdas et al. 2019; Leijon et al. 2002)*.* Information on occupation of the selected population concerns the year before participation in any of the SWES survey 2015 or 2017 and was obtained from the LISA database (Industry variable, branches, AstSNI2007G). We used the Swedish Standard Industrial Classification (Svensk Näringsgrensindelning 2007, SNI, Statistics Sweden, scb.se), which is based on the EU standard for classification of economic activities (Eurostat, NACE, Rev. 2, Statistical Classification of Economic Activities in the European Community 2008). According to the main activities and objectives of their workplaces, employees were in a first step classified into 15 larger employment branches (Table 1).

**Table 1:** Occupational classification of the study group

Type of occupation classified in occupations handling things, handling symbols and handling people based on industry category* and code (Code according to SNI, AstSNI2007G)	All	Men		Women	
		*n*	%	*n*	%
Handling “things”					
G01 Agriculture, forestry and fishing (A)	52	41	79	11	21
G02 Mining and quarrying, Manufacturing (B + C)	916	644	70	272	30
G03 Electricity, gas, steam and air conditioning supply, Water supply; sewerage, waste management and remediation activities (D + E)	116	87	75	29	25
G04 Construction (F)	366	319	87	47	13
G06 Transportation and storage (H)	340	251	74	89	26
Handling “symbols”					
G08 Information and communication (J)	315	215	68	100	32
G09 Financial and insurance activities (K)	164	67	41	97	59
G10 Real estate activities (L)	111	56	50	55	50
G11 Professional, scientific and technical activities (M) Administrative and support service activities (N)	717	365	51	352	49
G12 Public administration and defense; compulsory social security (O)	722	297	41	425	59
Handling “people”					
G13 Education (P)	1074	274	26	800	74
G14 Human health and social work activities (Q)	1337	212	16	1125	84

Number individuals and percent distribution of occupational categories related to industry category and sex (*n* = 6230)

The present study excluded three occupational categories: G05 Wholesale and retail trade *n* = 686, G07 Accommodation and food service activities *n* = 125, G15 Personal and cultural services *n* = 287

^*^Based on the LISA-variable for classification of occupations and branches in Sweden named AstSNI2007G, 15 categories (G01–G15), in accordance with EU standard (NACEREV2.0)

Three of the 15 original branches were excluded, as they were seen as too heterogeneous to be classified as to handling persons, things, or symbols. These were Wholesale and retail trade (G05), Accommodation and food service activities (G07), and Personal and cultural services (G15). The remaining 12 branches, which covers 85% of all employed in Sweden, were in the next step amalgamated into the three mentioned categories. The numbers and gender distribution of employed in the 12 branches and the chosen categorization into three occupational categories are shown in Table 1.

### Potential confounders

It is reasonable to believe that the employees’ prevalence of presenteeism and reasons for presenteeism can be affected by socio-demographic factors, physical and psychosocial work environment factors and health symptoms (Johns 2010; Keramat et al. 2020; Lohaus and Habermann 2019). The present study has used indicators on these factors, primarily as confounders when the association between occupational categories and reasons for presenteeism are analyzed, but they are also used in the descriptive section.

The socio-demographic factors, age, sex, level of education, sector of employment and income have been used. Age was classified into four age groups (16–29, 30–39, 40–49, and 50–64 years), and level of education in three categories referring to the highest completed education (compulsory or 9 years, high school or 12 years of education, and university education). Sector of employment was divided into three sectors, national public state sector, county and local council sector, and private sector. Income was measured through the concept annual disposable income in Swedish Crowns (10 SEK ≈ 1 Euro), which was available for the year preceding participation in the SWES survey. The annual disposable income is the personal income from work and public transfers minus current taxes and was classified in following three groups: low, < 250,000 SEK, medium 250,000–400,000 SEK, and high > 400,000 SEK. It should be noted that the variables education and income are seen as part of a hierarchical dimension and when used as confounders they reduce hierarchical differences between individuals within any of the three occupational categories. The information on all these socio-demographic variables was obtained from the LISA database.

The following two items from the SWES surveys were chosen as indicators of heavy physical work and strenuous work postures:

1. Does your job mean that your work is purely physical, i.e., do you put in more physical effort than you do when you walk, stand and move in the usual way? The six-point response scale (nearly all the time, about 3/4 of the time, 1/2 of the time, about 1/4 of the time, about 1/10 of the time, no, not at all) was dichotomized closest to the upper quartile to indicate the most adverse conditions. Yes (≥ 1/2 of the working time), No (≤ 1/4 of the working time).
2. Do you bend or twist yourself in your work in the same way repeatedly in an hour, for several hours during the same day?” The five-point response scale (every day, 1 day of 2, 1 day of 5, 1 day of 10, not at all) was dichotomized closest to the upper quartile to indicate the most adverse conditions. The dichotomized response alternatives are No (≤ 1 day of 5) and Yes (≥ 1 day of 2).

The following three items from the SWES surveys were chosen as the indicators of psychosocial work job demands, job control and job support:

1. Do you have so much work that you miss lunch, work late, or take work home? The five-point response scale (every day, 1 day of 2, 1 day of 5, 1 day of 10, not at all) was dichotomized closest to the upper quartile to indicate the most adverse conditions. The dichotomized response alternatives are Yes (≥ 1 day of 2) and No (≤ 1 day of 5).
2. Do you have the opportunity to determine your work pace?’’ The six-point response scale (nearly all the time, about 3/4 of the time, 1/2 of the time, about 1/4 of the time, about 1/10 of the time, no, not at all) was dichotomized closest to the upper quartile to indicate the most adverse conditions. The dichotomized response alternatives are No (≤ 1/10 of the time) and Yes (≥ 1/4 of the time).
3. Are you able to get support and encouragement from supervisors when work feels difficult?” Responses were given on a four-point scale (always, mostly, mostly not, never) and dichotomized into Yes (always, mostly) and No (mostly not, never)”.

Among available indicators of poor health in the SWES surveys, the following three most commonly reported items of health symptoms were selected:

- Have you experienced pain in your upper back or neck after work during the past 3 months?
- Have you had trouble sleeping during the last 3 months?
- Have you felt tired and listless during the last 3 months?

The five-point response scale (every day, 1 day of 2, 1 day of 5, 1 day of 10, not at all) was dichotomized closest to the upper quartile to indicate the most adverse conditions. The dichotomized response alternatives are No (≤ 1 day of 5) and Yes (≥ 1 day of 2).

### Statistical analyses

A cross sectional population-based cohort study was conducted. After investigating the distribution of the confounding variables and the frequencies of presenteeism and reported reasons for presenteeism for the three occupational categories, multiple logistic regression analyses were performed. Odds ratios (ORs) with 95% confidence intervals (CIs) for responding to any of the reasons for presenteeism were estimated for the occupational categories. The occupational category named ‘handling symbols’ was chosen as the reference category. Crude and adjusted models for the odds ratios for each of the reasons for presenteeism were calculated, where the adjusted model included control for all potential confounders (socio-demographic, working conditions and health symptoms).

In addition to these analyses, crude and adjusted odds ratios (ORs) were estimated for the five most often reported reasons for presenteeism related to the used confounders (age, sex, education, sector of employment, income*,* working condition and health factors*)*. These additional results are presented in supplementary file.

All statistical analyses were conducted with SAS, version 9.4, statistical software (SAS Institute, Inc., Cary, North Carolina).

## Results

In Table 2, the distribution of socio-demographic factors, work environment factors and health symptoms for the three occupational categories are presented. There were notable differences between the three occupational categories concerning age, gender distribution, level of education and employment sector. The category of employees handling things was dominated by men, had fewer employees with university education, and was dominated by employees working in the private sector. Employees handling symbols had a more even age and sex distribution, a relatively large share of university educated and was evenly distributed between privately and publicly employed. Employees handling people were predominantly female, often aged over 50, well educated, had the lowest share with high income and a large majority was employed by county and local council or public organizations (Table 2).

**Table 2:** Description of three occupational categories handling things, handling symbols, and handling people related to age, sex, level of education, sector of employment, income and health symptoms (2015 and 2017) (*n* = 6230)

	*n*	Handling things (%)	Handling symbols (%)	Handling people (%)
		*n* = 1780	*n* = 2029	*n* = 2411
Age (years)				
16–29	648	11.40	11.78	8.50
30–39	119	16.98	20.85	16.26
40–49	1674	25.98	30.31	24.64
50–64	2789	45.64	37.06	50.60
Sex				
Men	2828	74.97	49.29	20.16
Women	3402	25.03	50.71	79.84
Level of education				
University	3554	33.24	66.06	67.22
High school	2387	57.65	31.08	30.08
Compulsory	286	9.11	2.86	2.70
Employment sector				
National public state	832	6.93	26.37	7.18
County and local council	2310	5.42	18.14	76.52
Private	3088	87.65	55.50	16.30
Disposable income (SEK)				
0–250,000	1535	17.44	19.53	34.30
> 250,000–400,000	3430	61.43	50.30	54.38
> 400,000	1263	21.13	30.18	11.32
Work physically				
No (≤ 1/4 of time)	4806	66.97	89.82	75.38
Yes (≥ 1/2 of time)	1380	33.03	10.18	24.62
Strenuous postures				
No (≤ 1 day of 5)	4913	72.67	85.49	79.37
Yes (≥ 1 day of 2)	1272	27.33	14.51	20.63
High job demands				
No (≤ 1 day of 5)	4905	82.11	78.31	77.70
Yes (≥ 1 day of 2)	1291	17.89	21.69	22.30
Job control				
Yes (≥ 1/4 of time)	4814	81.84	84.47	69.07
No (≤ 1/10 of time)	1377	18.16	15.53	30.93
Job support				
Yes, always—mostly	4321	68.24	72.03	69.68
No, mostly not—never	1849	31.76	27.97	30.32
Upper back pain				
< 1 day of 10	3896	67.74	63.12	59.93
> 1 day of 5	2267	32.26	36.88	40.07
Tired and listless				
< 1 day of 10	3405	59.26	54.57	51.83
> 1 day of 5	2802	40.74	45.43	48.17
Sleeping troubles				
< 1 day of 10	3832	65.63	62.63	58.54
> 1 day of 5	2358	34.37	37.37	41.46

Table 2 additionally shows some differences between the occupational categories regarding work environment indicators. As expected, employees handling things more often than the other two categories reported heavy physical work and strenuous postures, but less often high psychosocial job demands. In contrast, employees handling symbols reported lower physical demands but higher psychosocial demand and higher job control than the other two categories. Employees handling people reported relatively high physical strain and high psychosocial demands, but also the highest share of low job control among the three categories (Table 2). The proportion reporting back symptoms, being tired and having sleeping troubles was somewhat higher among employees handling people when compared to the other two occupational categories.

The prevalence of presenteeism differed between the three occupational categories. Those employed in occupations handling things reported a prevalence of 35% of never having practiced presenteeism in the last year, employees handling symbols reported 30% and the figure for handling people was 26% (Table 3). The prevalence for having practiced presenteeism 2–3 times and four times or more was highest among employees handling people (Table 3). The most common reason for presenteeism given by employees handling things and employees handling symbols was ‘nobody else could take my responsibilities’ (38% and 50%, respectively), while the most common reason given by employees handling people was ‘I do not want to burden my colleagues’ (54%) (Table 3).

**Table 3:** Description of three occupational categories with varying work objects handling things, handling symbols, and handling people related to presenteeism and reasons for presenteeism (2015 and 2017) (*n* = 6230 and 4367)

	*n*	Handling things (%)	Handling symbols (%)	Handling people (%)
		*n* = 1780	*n* = 2029	*n* = 2411
Presenteeism (*n* = 6230)				
Never	1863	35.42	29.97	25.76
Once	1049	17.49	17.40	15.89
2–3 times	2100	31.17	33.22	36.00
4 times or more	1218	15.92	19.42	22.36
Reason for presenteeism, once or more (*n* = 4367)				
Because I do not want to burden my colleagues	1882	35.29	35.61	54.08
Because nobody else is able to carry out my responsibilities	1793	37.98	50.11	35.87
Because I enjoy my work	1287	31.31	32.51	25.87
Because I can’t afford taking sick leave	1045	20.50	21.32	28.21
Because I do not want to be considered lazy or unproductive	795	19.20	21.39	15.03
Because my pride depends on not taking sick leave	555	16.70	13.16	9.78
Because I am worried about being laid off	176	4.33	5.21	2.91
Because going to work was beneficial for my health	154	4.50	3.10	3.24
Because I want to maintain my social network	117	2.25	3.45	2.35
There were other reasons that I went to work	976	25.52	19.21	22.79

Reasons for presenteeism add up to more than 100 percent, because several reasons could be indicated

To understand the role of individual factors when other relevant factors were adjusted for, multiple regressions were carried out to estimate the odds for reporting a specific reason for presenteeism. The results of conducted regression analyses showed that the socio-demographic factors remained important in relationship to differences in reasons for presenteeism even after control for occupational sector (see supplementary file). Thus, older employees less often than younger reported ‘I do not want to be considered lazy’ (OR 0.18–0.44) and younger less often reported ‘I do not want to burden my colleagues’ (OR 0.61–0.77). Women more often than men reported ‘I do not want to burden my colleagues’ (OR 1.27), while men more often said ‘Nobody else can carry out my responsibilities’, but this difference was not statistically significant (see supplementary file). As expected those with lower income more often than those with higher income gave the reason ‘I can’t afford to take sick leave’ (OR 2.40–3.25), and those with university degree more often than those with lower education claimed that ‘Nobody else can carry out my responsibilities’ (OR 0.62–0.70) (see supplementary file). The answer ‘I do not want to burden my colleagues’ was more common among employees whose work was purely physical (OR 1.19) and among those with low job control (OR 1.59), while the answer ‘Nobody else can carry out my responsibilities’ was more common among those who reported high control (OR 0.66 and high demands (OR 2.49) (see supplementary file).

However, the large and systematic differences in reasons between the three occupational categories remained even after control for all potential confounders, including sex, age, income, health symptoms and the physical work environment factors (Table 4). In the response reflecting solidarity with colleagues, ‘I do not want to burden my colleagues’, the rate for employees handling people was elevated when compared to employees handling symbols (OR 1.68) (Table 4). A contrast was that reporting ‘I am worried about being laid off’ was less common among employees handling people than among employees handling symbols (OR 0.51). Among employees handling things the two reasons ‘Nobody else can carry out my responsibilities’ (OR 0.72) and ‘I can’t afford taking sick leave’ (OR 0.71) were less common when compared to employees handling symbols (Table 4). It should be noted, however, that there were no significant differences in odds ratios between the occupational categories in reporting “I enjoy my work”, “My pride depends on not taking sick leave”, “Going to work was beneficial for my health” and, “I want to maintain my social network” (Table 4).

**Table 4:** Crude and adjusted odds ratios (ORs) and 95% confidence intervals (CIs) for reported reasons for presenteeism by occupational category (*n* = 4367). Reference category ‘handling symbols’

Reason for presenteeism	*n*^a^	Handling things *n* = 1780			Handling people *n* = 2411		
		OR^b^	OR^c^	CI	OR^b^	OR^c^	CI
I do not want to burden my colleagues	1882	0.99	1.06	0.88–1.28	**2.13**	**1.68**	**1.40**–**2.02**
Nobody else can carry out my responsibilities	1739	**0.61**	**0.72**	**0.60**–**0.87**	**0.56**	0.93	0.77–1.13
I enjoy my work	1287	0.95	1.04	0.86–1.27	**0.72**	0.84	0.69–1.02
I can’t afford taking sick leave	1045	0.95	**0.71**	**0.56**–**0.89**	**1.45**	1.12	0.90–1.40
I do not want to be considered lazy	795	0.87	0.89	0.70–1.12	**0.65**	**0.77**	**0.61**–**0.98**
My pride depends on not taking sick leave	555	**1.32**	1.09	0.85–1.40	**0.72**	0.92	0.70–1.22
I am worried about being laid off	176	0.82	0.65	0.42–1.02	**0.55**	**0.51**	**0.31**–**0.82**
Going to work was beneficial for my health	154	1.47	1.06	0.67–1.69	1.05	1.22	0.74–2.02
I want to maintain my social network	117	0.64	0.78	0.45–1.34	0.67	0.58	0.34–1.00

The analyses are stratified and based on reasons for presenteeism, using multivariate logistic regression analyses, where ‘handling symbols’ was chosen as the reference category

^a^Number of individuals (*n*)

^b^Odds ratio (OR), and 95% confidence interval (CI), crude, for reason on presenteeism. Bold OR = statistical significant at the *p* < 0.05 level

^c^Odds ratio (OR), and 95% confidence interval (CI), adjusted for all potential confounders (representing socio-demographic, income, employment sector, working conditions, and health symptoms). Bold OR = statistical significant at the *p* < 0.05 level

## Discussion

The present study showed differences between the three occupational categories both with respect to prevalence of presenteeism and with respect to the different reasons the employees reported. There were also variations in reasons related to socio-economic background factors, work environment factors and health, but the differences between occupational categories remained mostly significant even after controlling for these factors. The largest contrast was found between employees handling people and employees handling things. The most common reason among employees handling people was ‘I do not want to burden my colleagues’ indicating solidarity with workmates but also that work in this sector is often team-oriented. Among employees handling symbols the most common answer was ‘Nobody else can carry out my responsibilities’, which may indicate a high degree of specialization in the occupations involved as well as individualized organization of work tasks and obligations.

The shown differences in given reasons for presenteeism between women and men, as well as between young and old are in agreement with other studies (Al Nuhait et al. 2017; Dudenhöffer et al. 2017; Gustafsson Senden et al. 2016; Hansen and Andersen 2008; Jena et al. 2012; Johansen et al. 2014; Rosvold and Bjertness 2001). One recent research review on presenteeism related to infectious illness, however, concluded that results on gender and age were inconsistent in cases of infectious disease (Webster et al. 2019). The found differences in the present study that presenteeism was related to employment sector and educational level have also been reported in one other study (Johansen et al. 2014). Other studies have found that the reasons for presenteeism also varies in relation to health differences (Bockerman and Laukkanen 2010), and income (Navarro et al. 2018; Skerjanc and Dodic Fikfak 2020; Webster et al. 2019). High psychosocial job demands and low supervisor support among different groups of employees could explain the differences in prevalence and reasons for presenteeism in the present study as well as in previous studies (Brborovic et al. 2017; Dudenhöffer et al. 2017; Hansen and Andersen 2008; Johns 2010; Nordenmark et al. 2019; Webster et al. 2019). No previous study has been found that could verify our results on the role of low control at work in explaining reasons for presenteeism.

The fact that differences between occupational categories in the prevalence and reasons for presenteeism remained even after confounder control is in line with research that has shown differences related to occupational hierarchies as well as to horizontal divides (Al Nuhait et al. 2017; Bracewell et al. 2010; Dudenhöffer et al. 2017; Johansen et al. 2014; McKevitt et al. 1997; Nordenmark et al. 2019). Interestingly, the gender differences remained in our study even after control for work sector. Independent of all sector belonging women more often than men motivated their presenteeism by “not wanting to burden colleagues”, while men more often referred to irreplaceability. As the occupations ‘handling people’ are women dominated and the occupations ‘handling things’ are male dominated the combination of gender and occupational sector makes the occupational difference even stronger.

The idea about presenteeism as being at least partly health promoting for the individual (Karanika-Murray and Biron 2020), does not get support in the present study. Only few respondents in all three occupational categories reported that going to work would be beneficial for their health. A larger share, particularly in the occupational category ‘handling people’, reported that they could not afford taking sick leave, and this may indicate that presenteeism is involuntary in these cases as Holland and Collins has shown in a study of employees with rheumatoid arthritis (Holland and Collins 2018). However, our study relies on self-reported reasons and presenteeism may well be beneficial although the individual does not experience presenteeism in this way.

In general, the interpretations of presenteeism in previous studies are linked to differences in professional cultures, although few have empirically studied differences in such cultures related to employees’ self-reported reasons. As Johns (Johns 2010) has pointed out, the social dynamics or collective motives of occupational categories that are part of professional cultures should include ‘the reaction of colleagues and clients to the act of presenteeism, both as encouragers and discouragers’. The idea behind the present study was that occupations classified according to their work objects would take this aspect into consideration. The fact that in occupations handling people, where teamwork and vulnerable clients are common, the main reason for presenteeism was solidarity with colleagues and clients illustrates the role of the character of the work object in these occupations. Also, the responses in occupations handling symbols may be seen to reflect the social dynamic as individualistic and specialized, and the reason for presenteeism often given is that nobody else can carry out the individual’ responsibilities point at similar motives. However, more studies are warranted on the role of shortage of resources, degree of specialization and management strategies for absence and presence.

### Strengths and limitations

The descriptive character of this cross-sectional study excludes any strong causal inferences, although the problem concerning reversed causality is limited, as the classification of occupational categories cannot reasonably be caused by employees’ reasons for presenteeism. The size and representativeness of the study group and the good quality of the measurements of prevalence of presenteeism and employees reported reasons for presenteeism are advantageous. The availability of data on socio-demographic factors, work environment factors and health are also valuable. As pointed out by Navarro and collaborators, the chosen low cut-off points for presenteeism of the present study may also be affected by the varying abilities among employees to choose sickness absence (Navarro et al. 2019). The fact that different studies have used different instruments in assessing presenteeism as well as self-reported reasons and that few of these have been assessed for measurement qualities restricts comparability between studies (Aboagye et al. 2020; Ospina et al. 2015). Another main limitation is the lack of substantive and empirically founded theories that may be able to interpret the results of the study, specifically related to the found differences between occupations based on the work object. Theories on professional cultures are still too vague and too much focused on professional ideals to capture the large differences between horizontally classified occupational categories.

## Conclusions

Differences between occupational categories with respect to the main work object seem to be important for the prevalence of presenteeism and the underlying reasons. As presenteeism has been shown to affect the future health of employees and the productivity of the work unit, attempts to reduce presenteeism may be important. In the case of infectious disease, presenteeism may also jeopardize the health of clients, patients or colleagues. Organizational measures including policies concerning job insecurity and sickness absence as well as improved work environments are warranted. As the reasons vary between occupations, specific measures should be applied in different occupational settings. Thus, among employees handling people it seems to be relevant to improve the system for covering up absence in often team-oriented work. In specialized functions among employees handling symbols and handling things the corresponding focus could be the introduction of shared responsibilities for specific tasks and obligations.

## Supplementary Information

Below is the link to the electronic supplementary material.

Supplementary file1 (DOCX 28 KB)

## Data Availability

The data of this study are available from Statistics Sweden. The data is not publicly available but can be used if an ethical permission is present.
