# Supplementary material for: Reasons for presenteeism in different occupational branches in Sweden: a population based cross-sectional study
Source: Int Arch Occup Environ Health. 2021 Apr 29;94(6):1385–95. doi: 10.1007/s00420-021-01701-2 (PMC8292261; doi:10.1007/s00420-021-01701-2)
Supplement: Supplementary file 1 — Supplementary file1 (DOCX 28 KB) [file 420_2021_1701_MOESM1_ESM.docx]

**Supplementary file.** Occupations categories, socio-demographic variables (age, sex, education*),* sector of employment, income*,* working condition and health factors and risk of presenteeism (once or more times) for the five most often reported reasons for presenteeism^a^, 2015 and 2017 (n=4367).

|  | I do not want to  burden my colleagues | | |  | Nobody else can carry out my responsibilities | | |  | I enjoy my work | | |  | I can’t afford taking sick leave | | |  | I do not want to be considered lazy | | |
| --- | --- | --- | --- | --- | --- | --- | --- | --- | --- | --- | --- | --- | --- | --- | --- | --- | --- | --- | --- |
|  | N^b^=1882 | | |  | N^b^=1793 | | |  | N^b^=1287 | | |  | N^b^ =1045 | | |  | N^b^ =795 | | |
|  | OR ^c^ | CI |  |  | OR ^c^ | CI |  |  | OR ^c^ | CI |  |  | OR ^c^ | CI |  |  | OR ^c^ | CI |  |
| **Occupations** |  |  |  |  |  |  |  |  |  |  |  |  |  |  |  |  |  |  |  |
| Symbols | 1.00 |  |  |  | 1.00 |  |  |  | 1.00 |  |  |  | 1.00 |  |  |  | 1.00 |  |  |
| Thing | 1.06 | 0.88 | 1.28 |  | **0.72** | **0.60** | **0.87** |  | 1.04 | 0.86 | 1.27 |  | **0.71** | **0.56** | **0.89** |  | 0.89 | 0.70 | 1.12 |
| People | **1.68** | **1.40** | **2.02** |  | 0.93 | 0.77 | 1.13 |  | 0.84 | 0.69 | 1.02 |  | 1.12 | 0.90 | 1.40 |  | **0.77** | **0.61** | **0.98** |
| **Sex** |  |  |  |  |  |  |  |  |  |  |  |  |  |  |  |  |  |  |  |
| Men | 1.00 |  |  |  | 1.00 |  |  |  | 1.00 |  |  |  | 1.00 |  |  |  | 1.00 |  |  |
| Women | **1.27** | **1.09** | **1.47** |  | 0.86 | 0.73 | 1.00 |  | 0.90 | 0.77 | 1.05 |  | 0.84 | 0.70 | 1.00 |  | 0.99 | 0.82 | 1.20 |
| **Level of education** |  |  |  |  |  |  |  |  |  |  |  |  |  |  |  |  |  |  |  |
| University/college | 1.00 |  |  |  | 1.00 |  |  |  | 1.00 |  |  |  | 1.00 |  |  |  | 1.00 |  |  |
| High School | 1.00 | 0.86 | 1.16 |  | **0.72** | **0.62** | **0.85** |  | 1.13 | 0.96 | 1.33 |  | **1.42** | **1.18** | **1.69** |  | 0.90 | 0.74 | 1.10 |
| Compusory | 1.07 | 0.75 | 1.52 |  | **0.60** | **0.40** | **0.88** |  | 0.84 | 0.56 | 1.25 |  | **1.56** | **1.06** | **2.29** |  | **1.66** | **1.08** | **2.50** |
| **Age (years)** |  |  |  |  |  |  |  |  |  |  |  |  |  |  |  |  |  |  |  |
| 16–29 | 1.00 |  |  |  | 1.00 |  |  |  | 1.00 |  |  |  | 1.00 |  |  |  | 1.00 |  |  |
| 30–39 | **0.77** | **0.60** | **0.98** |  | 0.93 | 0.72 | 1.21 |  | 0.91 | 0.70 | 1.19 |  | 0.83 | 0.63 | 1.09 |  | **0.44** | **0.34** | **0.58** |
| 40–49 | **0.72** | **0.56** | **0.91** |  | 0.90 | 0.70 | 1.16 |  | 0.80 | 0.62 | 1.04 |  | 0.89 | 0.68 | 1.16 |  | **0.34** | **0.26** | **0.45** |
| 50–64 | **0.61** | **0.49** | **0.77** |  | 0.79 | 0.62 | 1.01 |  | 0.78 | 0.61 | 1.01 |  | **0.76** | **0.59** | **0.99** |  | **0.18** | **0.14** | **0.23** |
| **Employment sector** |  |  |  |  |  |  |  |  |  |  |  |  |  |  |  |  |  |  |  |
| National public state | 1.00 |  |  |  | 1.00 |  |  |  | 1.00 |  |  |  | 1.00 |  |  |  | 1.00 |  |  |
| County and local council | 0.93 | 0.74 | 1.16 |  | 0.82 | 0.65 | 1.03 |  | 0.82 | 0.64 | 1.04 |  | 0.84 | 0.65 | 1.10 |  | 0.75 | 0.56 | 1.00 |
| Private | **0.81** | **0.66** | **0.99** |  | **1.29** | **1.05** | **1.59** |  | **0.73** | **0.59** | **0.91** |  | **0.67** | **0.53** | **0.86** |  | 1.02 | 0.79 | 1.32 |
| **Disposabel income** |  |  |  |  |  |  |  |  |  |  |  |  |  |  |  |  |  |  |  |
| >400 000 SEK | 1.00 |  |  |  | 1.00 |  |  |  | 1.00 |  |  |  | 1.00 |  |  |  | 1.00 |  |  |
| >250 000–400 000 SEK | **1.43** | **1.19** | **1.71** |  | **0.62** | **0.52** | **0.74** |  | 0.92 | 0.77 | 1.11 |  | **2.40** | **1.85** | **3.16** |  | 1.03 | 0.81 | 1.30 |
| 0–250 000 SEK | **1.55** | **1.24** | **1.94** |  | **0.42** | **0.33** | **0.53** |  | **0.76** | **0.60** | **0.97** |  | **3.25** | **2.42** | **4.41** |  | 1.31 | 0.99 | 1.75 |
| **Work physical,** Yes ( > 1/2 of time) | **1.19** | **1.00** | **1.42** |  | **0.53** | **0.43** | **0.64** |  | 0.98 | 0.81 | 1.20 |  | **1.42** | **1.17** | **1.72** |  | 0.96 | 0.77 | 1.21 |
| **Strenuous work**, Yes (> 1 day of 5) | 0.95 | 0.80 | 1.13 |  | **0.81** | **0.67** | **0.97** |  | **0.78** | **0.64** | **0.95** |  | **1.54** | **1.28** | **1.85** |  | 1.01 | 0.81 | 1.25 |
| **Job demands**, Yes (> 1 day of 5) | **1.17** | **1.00** | **1.37** |  | **2.49** | **2.13** | **2.92** |  | **1.22** | **1.03** | **1.45** |  | **0.80** | **0.66** | **0.97** |  | 0.91 | 0.75 | 1.11 |
| **Job control,** No (< 1/10 of time) | **1.59** | **1.37** | **1.86** |  | **0.66** | **0.56** | **0.78** |  | **0.67** | **0.55** | **0.80** |  | 1.11 | 0.93 | 1.32 |  | **1.36** | **1.12** | **1.65** |
| **Job support,**  No (mostly not—never) | 1.03 | 0.90 | 1.19 |  | **1.28** | **1.10** | **1.48** |  | **0.55** | **0.47** | **0.64** |  | **1.25** | **1.06** | **1.47** |  | 1.18 | 0.99 | 1.41 |
| **Upper back pain** (> 1 day of 5) | 0.98 | 0.85 | 1.13 |  | 1.05 | 0.91 | 1.22 |  | 0.88 | 0.76 | 1.02 |  | **1.27** | **1.08** | **1.50** |  | **1.21** | **1.02** | **1.45** |
| **Tired and listless** (> 1 day of 5) | **1.22** | **1.05** | **1.41** |  | 1.02 | 0.87 | 1.19 |  | **0.65** | **0.56** | **0.76** |  | **1.39** | **1.17** | **1.66** |  | **1.55** | **1.28** | **1.89** |
| **Sleeping troubles** (> 1 day of 5) | 1.06 | 0.92 | 1.22 |  | **1.16** | **1.00** | **1.34** |  | 1.02 | 0.87 | 1.19 |  | **1.35** | **1.14** | **1.59** |  | **1.33** | **1.11** | **1.59** |

^a^ The analyses are stratified and based on five reasons for presenteeism, using multivariate logistic regression analyses. ^b^ Number of individuals (n).  ^c^ Odds ratio (OR), and 95% confidence interval (CI), adjusted for all exposure variables and confounders representing socio-demographic characteristics and working conditions, including health symptoms. Bold OR=statistical significant at the p< 0.05 level.
